# Report of a Case of Otitic Brain Abscess, with Remarks on Diagnosis1Read before the Buffalo Academy of Medicine, February 4, 1896.

**Published:** 1896-05

**Authors:** Alvin A. Hubbell

**Affiliations:** Buffalo, N. Y., Professor of ophthalmology and otology, Medical Department of Niagara University; 212 Franklin Street


### REPORT OF A CASE OF OTITIC BRAIN ABSCESS, WITH REMARKS ON DIAGNOSIS.[1]

By ALVIN A. HUBBELL, M. D., Buffalo, N. Y.,
Professor of ophthalmology and otology, Medical Department of Niagara University.

WILLIAM B., aged 20, born in the United States, occupation, waiter; entered Erie County Hospital, July 22, 1895, with a history of having been indisposed for some time previously with headache, loss of appetite and nausea. The left ear had discharged since he was six years of age, but from what cause he did not know. He did not remember having had measles, scarlet fever or diphtheria. During the last three days he has had severe pain in the left ear and left side of the head, and had been unable to take food or to sleep. Upon examination a polypus was found nearly filling the left auditory canal, and there was also a considerable and offensive discharge from this ear. He complained of soreness and increased pain when the ear was handled, but there was no swelling or tenderness over the mastoid. At that time and on the day following, I regarded the case as one of acute otitis, supervening upon the chronic otorrhea.

On July 23d, I removed the larger portion of the polypus with a snare and ordered the ear to be gently syringed with warm antiseptic solutions three or four times a day, and the patient kept in bed.

July 25th.—The auditory canal was somewhat swollen and painful and the fetid discharge continued. The head-symptoms had become more pronounced and the patient seemed dull and restless. The pulse was 60 and full; respiration, 10; temperature, 97; pupils reacted slowly to light. I expressed myself as being suspicious of an intracranial, probably cerebral, abscess. An ice bag was ordered to be applied to the side of the head and around the ear, and the ear syringed with warm boracic acid solution, to be followed by instillations of diluted alcohol twice a day.

July 26th.—Patient has increased pain in the head and ear and has been very restless; pulse, 63; temperature, 99½; respiration, 32.

1. Read before the Buffalo Academy of Medicine, February 4, 1896.

Ordered opiates to relieve pain. My diagnosis of cerebral abscess was quite positive and I requested the attending neurologist and surgeon to see the case the next morning with a view to an operation, should they coincide with me in the diagnosis.

July 27th.—Drs. Putnam and E. A. Smith saw the case this morning and viewed the symptoms in somewhat different light from what I had and did not advise an operation. In the afternoon I found that the patient had had a convulsion. Pulse was 50; temperature, 97; respiration, 10; face somewhat livid. Stimulants were given, counter-irritants applied to the back of his neck and his feet put in hot mustard water.

July 28th.—At 2 A. M. patient had another convulsion, pulse went down to 32 and the respiration was 4 or 5 per minute. The patient was given stimulants freely and he rallied from what was feared to be a moribund condition. Previous to this convulsion for twenty-four hours, the pulse had ranged from 60 to 70 and the temperature from 100 1-5 to 101 1-5, and the respiration from 16 to 20. At 8 A. M. the pulse had risen to 70, temperature, 101 3-5, and respiration, 18. At noon the pulse was 74; temperature, 100 4-5; respiration, 20. At 2.30 P. M. patient had another convulsion. Stimulants were again given and counter-irritants applied to the back of the neck. He slept quietly for three hours. At 6 P. M. he vomited a greenish fluid. At 8 P. M. the pulse was 80; temperature, 101. At midnight pulse was 110; temperature, 101 4-5. He was delirious all night.

July 29th.—Another consultation was held, there being present Drs. E. A. Smith, Tremaine, Putnam and myself. The patient was then in a comatose state and was rapidly sinking. The pulse had become very rapid and weak and the temperature had risen to 103 3-5 and the respirations to 30. My diagnosis of celebral abscess located in the tempor-sphenoidal lobe of the brain was presented to my consultants and my reasons for it explained. The patient's condition, however, was so extreme that any operation for exploratory or therapeutic purposes was agreed to be inadvisable. At 4 P. M. the patient died.

By way of recapitulation I may add to the above hospital record, which in some respects is not as full as it might be, that the patient came into the hospital very sick. He had had pain in his left ear and left-sided headache for two or three weeks, with loss of appetite, nausea, history of chilliness with some fever and deprivation of sleep. He soon showed the characteristic mental sluggishness and finally delirium, the chills, vomiting, dizziness, prostration, convulsions, and the normal, slightly subnormal, or moderately elevated temperature, the slow full pulse, slow respiration, together with the history of otorrhea, polypus of the middle ear, acute ear-symptoms, developing on the chronic, and the significant pain in the ear and head. There were no symptoms or history of mastoid disease at any time. Judging by the results of the post mortem examination, the time required for the formation of such an amount of pus must have far exceeded the seven days that he was in the hospital. The disease must have been in progress in the brain for two or three weeks before his admission.

Post mortem examination was made July 31st, by Dr. H. U. Williams, who gives me the following report:

The convexity of the brain shows recent acute fibrinous leptomeningitis. The same condition existed in a more marked degree at the base, involving the surface of the pons and medulla from the basilar arteries, etc., reaching anteriorly to the optic chiasm. Moderately firm adhesion on the left side fastened the tempor-sphenoidal lobe to the upper border of the petrous portion of the temporal bone, one-half inch from the squamous portion, around which adhesion was abundant yellowish lymph. Opposite these adhesions was a cavity in the tempor-sphenoidal lobe, the size of a walnut, lined by grayish necrotic tissue and containing pus with shreds of grayish necrotic tissue. This cavity connected with the middle ear by two or three distinct openings through the roof of the latter.

I was present at the post mortem examination, and I will add to the statement of Dr. Williams that the bone immediately over the tympanic cavity was denuded of its periosteum as well as perforated by a number of small openings. (Brain exhibited.)

### REMARKS.

This case is quite a typical one of cerebral abscess, and I offer it as an introduction to what I have to say on this important subject.

It was, comparatively, but yesterday since brain abscess was regarded at all as a curable disease. Before this, the prognosis, especially in the otitic form, was very unfavorable, almost every case terminating fatally. But in 1886, when Dr. Truckenbrod[1] reported a case operated upon by Dr. Schede, of Hamburg, Germany, and which was cured, and Mr. Arthur E. Barker, of London, for Dr. W. E. Gowers[2] trephined for cerebral abscess of otitic origin, the patient recovering, a new impetus and hope was given to the treatment of this disease. In view of the results obtained by surgical measures, the diagnosis becomes of supreme interest and of greatest concern.

FREQUENCY AND RESULTS.

As the literature of the disease shows, and especially during the past ten years, when surgery has greatly augmented the statistics, brain abscess of otitic origin is found to be by no means an extremely rare affection. Within my own observation, during the past six years, I can recall at least eight undoubted cases, in two of which the diagnosis was confirmed by post mortem examinations. Nearly all experienced practitioners probably could bear similar testimony. Many cases of brain disease, furthermore, have been treated heretofore, and have died, that were very probably otitic in origin, but the etiology had been overlooked. Arthur E. Barker,[3] of London, found that in ten years (1878–87 inclusive,) according to the registrar-general's reports, 3,570 had died from "otorrhea." He adds: "If it were possible to sift all these latter groups ('simple meningitis,' 'unclassified brain disease' and 'general septic affections') and to rearrange them according to the primary cause of death, there can be no doubt that these annual 400 deaths, attributed to ear disease, would be swelled to four or five times the number." Dr. G. Newton Pitt,[4] of London, in an analysis of 57 autopsies of cases in which ear trouble had set up brain disease, found that they were from a total of 9,000 autopsies, showing 0.66 per cent. "Further examination shows," he says, "that 27 of the cases had occurred during the last four years, which would raise the ratio for that period to 2 per cent." Dr. Thomas Barr,[5] of Glasgow, found that in London, in 1882, there were 86 deaths attributed to otorrhea, and in eight principal towns of Scotland the number for one year was 26. He believes these numbers, "considerable as they are, do not express anything like the real number of victims of this disease who perish annually" in those places.

In 1892, Dr. Frank Allport,[6] of Minneapolis, Minn., collected 169 cases, mostly reported since 1879, each of which had had "an ear difficulty resulting in intracranial trouble, death, and an autopsy," or had had "an ear difficulty resulting in intracranial trouble, and the intracranial cavity had been exposed by operation."

I might add much more and similar testimony in this direction, but the above is amply sufficient to show that intracranial disease arising from otorrhea is not a rare affection. Moreover, the otorrhea need not necessarily be chronic in its nature, as Eulenstein[7] has recently reported 18 cases of cerebral abscess developing after acute inflammation of the middle ear or temporal bone, and Allport[6] has reported 10 cases. Cerebral disease following ear disease has been found to be so frequent that most insurance companies regard suppuration of the middle ear as a sufficient cause for the rejection of an applicant affected by it.

Ear suppuration does not always lead to the same disease of the brain, or to disease in a particular part of the brain. For example, the intracranial affection may be phlebitis and thrombosis of the large sinuses, or meningitis, or cerebritis, with or without suppuration.

If brain abscess develop, it may be within the dura mater, or in almost any part of the cerebrum or the cerebellum or even in the pons varolii. Dr. G. Newton Pitt[8] says: "Cerebral abscesses occur rather less frequently than meningitis or thrombosis." Allport,[9] in the 169 cases collected of otitic brain disease, found 98 of them abscesses proper. Dr. Macewen,[10] of Glasgow, has reported 94 cases of intracranial lesions from ear disease, of which 25 were abscesses of the brain and 5 extradural abscesses.

In the majority of cases these abscesses are near that part of the ear from which the infection proceeds. Dr. Pitt[11] says: "In two-thirds of the cases the cerebral abscesses occur close to the roof of the tympanum." In another place he says:[12] "These abscesses are almost always situated very close to the roof of the tympanum. . . . . Cerebellar abscesses are less common and will probably be associated with disease of the dura mater, behind the petrous bone."

The position of the brain abscess, according to Von Bergman,[31] is always a typical one, and is either in the temporal lobe or in the side of the cerebellum corresponding to the diseased side. Dr. Thomas Barr,[14] of Glasgow, has analysed 76 cases of brain abscess, and found it located in the cerebrum (temporal lobe) in 55 cases; in the cerebellum, in 13; in both, in 4; in the pons varolii, in 2; in the crus cerebelli, in 1. Körner,[15] in 100 cases of brain abscess, found it 62 times in the cerebrum, 32 times in the cerebellum and 6 times in both.

When the abscesses are in the cerebrum they are, according to A. E. Barker,[16] situated "almost invariably in the temporal lobe, behind a line drawn vertically through the tragus. From this they may extend backward into the occipital lobe to a greater or less extent, and very rarely forward toward the apex of the temporal lobe. But these exceptions are so uncommon that they need hardly to be considered for practical purposes." In the cases of cerebellar abscesses we find them, he says, "almost invariably seated in the anterior portion of the lateral lobes, where these lie in contact with the posterior surface of the petrous bone and lateral sinus."

Macewen,[17] in 17 cerebral abscesses, found them in the temporosphenoidal lobe 10 times; in the frontal, 2; in the parietal, 1; "superficial" (ulceration of brain), 4.

So much for the frequency and situation of otitic brain abscess. As to results of treatment, it may be said that before 1886 it was well-nigh hopeless, unless, perchance, a spontaneous opening and evacuation took place. In 1885, Schede,[18] after having chiselled open the mastoid, found, two weeks afterward, symptoms of brain abscess, and by these symptoms, the great edema, above and behind the mastoid, and a fistula extending upward and beneath the periosteum into diseased bone, was led to extend his former opening by chiselling, until the abscess in the brain was reached and evacuated. In the same year A. E. Barker[19] made the more signal innovation of proceeding at once to find and evacuate a cerebral abscess, acting alone upon the evidence of the diagnosis, "quite independently of visible or tangible change, beyond a slight moisture of the middle ear, there being no evidence whatever, externally, as to what was going on within the skull."[20] The reports of these two cases gave a wonderful impetus to the surgical treatment of brain abscess. What have been some of the results? In 1893, Heiman[21] reported 32 cases operated upon, 17 of them recovering. Von Beck[22] has collected 36 cases that have been operated upon, including 29 in the temporal lobe, of which 17 of the latter were cured. Körner[23] says: "Of the 55 abscesses which thus far have been evacuated, 29 ended in recovery, 26 in death." Macewen[24] published, in 1893, 25 cases of brain abscess, of which 19 were operated upon, and all but one recovered. Dr. Barr,[25] of Glasgow, in his address at the International Otological Congress, held in Florence the past year (1895), said that at least 59 cases of cerebral and 7 cases of cerebellar abscess had been successfully treated.

Such results as these, in otherwise hopeless cases, mark a surgical triumph and a saving of life that compel a study of diagnosis, and a determination of pathological conditions in this direction, never before so imperative as now, on the part of all practitioners of medicine. Von Bergman[26] has forcibly said: "Whoever knows, as we do, that there is no other termination to cerebral suppuration than a fatal outbreak into the ventricles, or equally fatal meningitis, must look upon the surgical opening of cerebral abscesses as the rescue from an urgent danger to life.'' Sahli[27] adds: "In this province much has already been done, and, with increased refinement in diagnosis, much more remains to be accomplished."

My remarks, so far, are intended to lay stress upon the frequency with which otorrhea leads to brain abscess, together with its most probable situation, and to bring prominently into view the rich fruitage yielded by surgical interference; and all this culminates in a tremendous urgency of correct diagnosis.

It should be borne in mind that brain abscess from ear disease is not always a simple, isolated process, but is often associated with meningitis, thrombosis and mastoiditis. When these or other associated affections are present, the symptoms of abscess may be confused and masked. Hence, in restricting myself here to the diagnosis of brain abscess alone, the possibility of the coëxistence of other diseases should not be forgotten.

### DIAGNOSIS.

The progress of brain abscess may be divided into the initiatory, established and terminal stages.

In the *initiatory stage* there is a history of otorrhea, generally chronic in character, with discharge, slight or profuse, fetid, and oftentimes there are present one or more polypi in the external auditory canal. Following some injury or exposure, acute inflammatory symptoms arise in the ear or mastoid, characterised by tenderness and swelling about and behind the ear, and pain in the ear or in the temporal region, or indefinitely extending with more or less severity over the corresponding side of the head, and sometimes to both sides of the head. The discharge of the ear at this time often diminishes or ceases. Chilliness or pronounced rigors may be felt and there may be slight or markèd elevation of temperature. The patient loses appetite, sometimes has nausea, and is sleepless and restless from the pain. The pulse is increased in frequency, the tongue becomes coated and there is general weakness and prostration. This stage lasts from two to ten days and the symptoms gradually merge into those indicating the development of encephalic disease.

When intracranial disease is *established*, the first symptoms are essentially those of encephalic inflammation. The previous headache is increased and often becomes excruciating, and extends from the side of the diseased ear to all portions, even to both sides of the head and down the back. There is restlessness, nausea and sometimes vomiting, chilliness, temperature raised from 100° to 102°, and variable. There is thirst, continued loss of appetite, and constipation. There may or may not be tenderness on pressure of the affected side. This inflammatory period extends for a number of days and is followed by symptoms depending upon suppuration and intracranial pressure. Those depending upon suppuration are the same as those found in any suppuration—namely, chills, slight or severe, febrile manifestations with a moderate rise of temperature, perhaps not as high as before, with sweating and weakness, dizziness, and the disturbances of the stomach may also continue. These symptoms may last indefinitely, for several days to two or three weeks, when those of pressure appear. Then, the headache may be slight or at times severe, and may become fixed and correspond to the location of the abscess, although it is more apt to be distributed over the entire head and, as I have seen, even extend into the spine. This headache is increased by muscular exertion, by a low position of the head, or by acceleration of the circulation from increased fever or use of stimulants. Percussion over the side of the head affected increases the pain. The dizziness is usually increased by change of posture, and if the middle lobe of the cerebellum becomes affected there may be unsteadiness of gait. Vomiting and derangements of the digestive organs may be increased, with occasional diarrhea. To these are superadded mental irritability, and the mind becomes dull, with slowness of thought, imperfect attention, impaired memory, occasional delirium, all increasing as the pressure in the cranial cavity increases, till there is great prostration, continuous delirium or stupor. The pulse is slower than at first, ranging from 50 to 65, and the temperature fluctuates around the normal line, sometimes being a little below, sometimes a little above. The respirations are also less frequent than normal and may approach the Cheyne-Stokes form in cerebellar abscess. Late in the disease, however, the pulse and respirations become more frequent and the temperature rises. The patient may continue to be chilly at times, but pronounced rigors are rare. Optic neuritis is frequent, especially in the later periods of the disease, and is not peculiar to any special situation of the abscess. When the abscess has so extended as to encroach upon the motor area of the cortex, convulsive movements or convulsions may take place. When reaching by disintegration or pressure to the motor tracts, there may be paralysis, which is chiefly hemiplegic, though incomplete, and is on the side of the body opposite the side of the head affected. It frequently follows unilateral convulsions, and it may be limited to the arm, with or without implication of the face. If the occipital lobe becomes involved, there may be hemianopsia, and encroachment upon the left frontal lobe may cause aphasia. Paralysis of the facial and auditory nerves may arise from disease of the temporal bone, and in such a case is always found on the side of the affected ear, and is usually incomplete.

Paralysis of the third cranial nerve on the same side as the lesion occurs in some cases of large abscess in the temporal lobe, and is shown by ptosis, divergent strabismus, indeed by paralysis of all the ocular muscles, except the external rectus and superior oblique, with dilatation of the pupil. Nystagmus, inequality in the size of the pupils, sluggish pupillary reaction to light, change of deep and superficial reflexes, peculiar fetid odor to the breath, "grayish" color to the face, and bodily emaciation may be noticed, but they are not of special diagnostic value. Dr. Macewen[28] describes the "elicitation of a differential cranial percussion note," but if such "note" exists, it requires a nicety of technique and discrimination to make it apparent that few can attain. It is not, therefore, of special importance in the hands of most practitioners.

The *terminal stage* of brain abscess, when allowed to pursue its course without surgical interference, ends, with very rare exceptions, in death. It is marked by symptoms of increasing pressure, with deepening stupor and coma, and by those caused by oozing of pus or rupture of the abscess into the ventricles or on to the surface of the brain. The latter excites fresh irritation and a rapidly spreading inflammation follows. This is generally indicated by the recurrence of "vomiting, restlessness, temporary squinting, flushing and erratic rigidity of the limbs, clonic spasms, trepidation and prostration, the pulse meanwhile becoming quick, the breathing hurried and the temperature high."[29]

If the abscess bursts into the ventricles, the symptoms appear more abruptly and are very marked, the whole aspect of the patient changing with great rapidity. The pupils become dilated, face livid, respiration hurried and perhaps stertorous, temperature rises to 104° or 105°, the pulse becomes quick and small, muscles twitch, or perhaps there are convulsions, and coma and death speedily follow.

Brain abscess is almost always acute in its course, but so-called latent or chronic cases have been recorded. It is possible that an abscess may become encysted. The duration, therefore, of the existence of an abscess varies within wide limits.

Spontaneous cures have been recorded, but they need not be looked for, and I need not consider them here.

### SUMMARY AND CONCLUSION.

To summarise, then, the diagnosis of brain abscess is made probable by:

1. The history of the case and of a preceding otorrhea and ear trouble.

2. Those general symptoms pointing to inflammation in the cranial cavity—namely, headache; nausea; vomiting; chilliness or chills; moderate elevation of temperature.

3. Those general symptoms indicating suppuration: Headache; chills; temperature a little elevated; nausea and sometimes vomiting; dizziness; optic neuritis, not always; followed by

4. Those symptoms caused by pressure from accumulation of pus: Mental sluggishness; temperature, not high, occasionally sub-normal; respirations, less frequent; pulse, slower (50 to 65); prostration; stupor; delirium.

5. Those localising symptoms of temporo-sphenoidal abscess: Convulsions, if the cortical motor area is involved or pressed upon; aphasia, if the speech center is reached in the left frontal lobe; hemianopsia, if the occipital lobe becomes affected; hemiplegia (opposite side and generally partial), if the internal capsule of the brain is pressed upon or involved, or if the cortical motor centers are destroyed; paralysis of the third nerve of the affected side, if contiguous pressure and meningitis are sufficient.

6. Those localising symptoms, if the abscess is cerebellar: Cerebellar incoördination, if there is pressure or involvement of the middle lobe of the cerebellum; persistent occipital pain, in some cases.

In studying and analysing a given case of otitic brain abscess, the differentiation must be made between it in its different stages, and acute purulent otitis or mastoiditis, meningitis, thrombo-phlebitis of the large brain sinuses, extradural collections of pus, and brain tumor, the symptoms of which I must pass over at this time.

Most writers regard the diagnosis of brain abscess as difficult, while others seem to view it as comparatively easy and certain.

Heiman,[30] of Warsaw, says: "In three-fourths of the cases of otitic brain abscess the course is such that a diagnosis can be made.

The careful weighing of all the symptoms, the localisation of the bony affection (temporal bone, A. A. H.), the etiological elements, and the length of time the patient has been under observation, if carefully considered, enable us not only to diagnosticate the abscess, but often to locate it." Dr. Barr[31] says "that an uncomplicated abscess in the temporo-sphenoidal lobe may be said now to belong to the region of certainty."

Should there, however, remain doubts as to the existence of abscess after earnest and careful study of suspicious symptoms, an exploratory operation should be performed and search made with aspirator for the presence of pus. If pus is found, the operation may be extended so as to evacuate it; and if not found, the wound may be closed without jeopardising the life of the patient. In three cases I have seen the exploratory operation made without finding pus and without shortening the life of the patients.

Von Bergman[32] in his monograph upon the surgical treatment of intracranial disease (Hirnkrankheiten), asserts that the history of otorrhea, past or present, together with persistent sleeplessness and a temperature remaining steadily at about 99°, are sufficient indications for opening the cranial cavity for the purpose of exploration.

Let me finally urge that these considerations in regard to the diagnosis of brain abscess be pressed home to every one of us, and stimulate us to a judicious and timely resort to those surgical measures which, today, promise so much in this disease.

---

1. Archives of Otology, Vol. XV., 1886, page 177.
2. British Medical Journal, Dec. 11, 1886, page 1154.
3. Hunterian Lectures on Intracranial Inflammation, etc., delivered June, 1889, reprint page 3.
4. Glustonian Lectures on Some Cerebral Lesions, British Medical Journal, March 22, 1890, page 643.
5. British Medical Journal, April 2, 1887, page 724.
6. Journal of the American Medical Association, 1892, Vol. XIX., numbers 16 to 26 inclusive, page 471.
7. Monatsschrift für Ohrenheilkunde, No. 3, 1895; also Archives of Otology, Vol. XXIV., 1895, page 377.
8. Glustonian Lectures on Some Cerebral Lesions, British Medical Journal, April 5, 1890, page 772.
9. Journal American Medical Association, December 10, 1892, page 691.
10. Pyogenic Infective Diseases of the Brain and Spinal Cord, 1893, page 332.
11. British Medical Journal, April 5, 1890, page 772.
12. Loc. Cit., March 22, 1890, page 646.
13. Surgical Treatment of Brain Abscess, Archives f. Klin. Chir., Vol. XXVI.; also Politzer on Diseases of the Ear, 1894, page 473.
14. Abscess in the Brain, Resulting from Disease of the Ear, British Medical Journal, April 2, 1887, page 724.
15. Archives f. Ohrenheilkunde, Vol. XXIX.; also Archives of Otology, Vol. XX., 1891, page 81.

16. Hunterian Lectures on Intracranial Inflammation, etc., 1889, page 35 and page 38.
17. Loc. Cit., page 333
18. Archives of Otology, Vol. XV., 1886, page 176.
19. British Medical Journal, December 11, 1886.
20. British Medical Journal, March 5, 1887, page 538.
21. Archives of Otology, 1893, Vol. XXII., page 60.
22. Beit. z. Klin. Chirurgie, Vol. XII., No. 1; also Archives of Otology, Vol. XXIV., 1895, page 91.
23. Die Otitischen Erkrankungen des Hirns, etc., 1894; also Archives of Otology, Vol. XXIII., 1894, page 144.
24. Loc. Cit., page 333.
25. Archives of Otology, Vol. XXIV., 1895, page 309.
26. Truckenbrod, Archives of Otology, Vol. XXI., 1892, page 184.
27. Ibid, page 184.
28. Loc. Cit., p. 146.
29. Macewen, loc. cit., p. 143.
30. Archives of Otology, 1893, Vol. XXII., p. 55.
31. Archives of Otology, Vol. XXIV., 1895, p. 307.
32. Dench, Diseases of the Ear, 1894, p. 44.

212 Franklin Street.

